# Supplementary material for: Endothelial cell specific molecule 1 promotes epithelial-mesenchymal transition of cervical cancer via the E-box binding homeobox 1
Source: PLoS One. 2024 Jul 2;19(7):e0304597. doi: 10.1371/journal.pone.0304597 (PMC11218952; doi:10.1371/journal.pone.0304597)
Supplement: Attachment — Submitted filename: Response to Reviewers.docx [file pone.0304597.s002.docx]

**6. Review Comments to the Author**

**Reviewer #1: The author did not securely answer the question about the adequacy of the validation of the conclusions, suggesting an in-depth study before resubmitting it for review.**

Dear reviewers and editor:

Thank you for your insightful comments and for the opportunity to enhance the quality of our manuscript. We appreciate your concern regarding the adequacy of the validation of our conclusions.

In response to your suggestion for a more in-depth study, we would like to clarify the validation methods employed in our research, which may not have been sufficiently detailed in our initial submission. Our study utilized a comprehensive approach combining bioinformatics analysis, molecular docking, immunohistochemistry, immunofluorescence, and in vivo experiments to validate the role of ESM1 in cervical cancer progression and the therapeutic potential of aloe-emodin (AE).

1. Bioinformatics Analysis: We employed databases such as the Human Protein Atlas, LinkedOmics, and GEPIA to substantiate the expression and prognostic significance of ESM1 in cervical cancer. These analyses provided a robust statistical foundation for ESM1's role in cancer biology.

2. Molecular Docking: Using Schrodinger software, we performed molecular docking studies to predict the interaction between AE and the ESM1 protein. This provided molecular-level evidence supporting the potential of AE as a targeted therapy.

3. Immunohistochemistry and Immunofluorescence: These methods were crucial in validating the expression levels of ESM1 and the impact of AE treatment on EMT markers in tissue samples and cell lines, respectively.

4. In Vivo Experiments: The use of a cervical cancer mouse model allowed us to observe the effects of AE on tumor growth and metastasis, providing compelling evidence of its therapeutic potential.

We agree with your suggestion for further in-depth research. In future studies, we will focus on the following aspects:

1. Extended Clinical Data Analysis: We plan to collaborate with clinical partners to access a larger cohort of patient samples to reinforce our findings.

2. Long-term Efficacy and Safety Studies: Conducting long-term treatment studies to observe the prolonged effects and potential resistance mechanisms of cervical cancer.

3. Mechanistic Studies: Further investigations into the molecular mechanisms by which AE modulates ESM1 and its downstream signaling pathways.

We believe that these additional studies will provide a more comprehensive understanding of the therapeutic potential of AE and firmly establish the validity of our conclusions. We are committed to conducting these studies and updating our manuscript accordingly. We will also include the aforementioned research directions, or the limitations of this study in the Discussion section marked in blue color.

Thank you once again for your constructive feedback. We hope that our response and proposed additional studies adequately address your concerns and demonstrate our commitment to rigorous scientific inquiry.

Sincerely,

Sufen
